# Supplementary material for: Exertional Exhaustion (Post-Exertional Malaise, PEM) Evaluated by the Effects of Exercise on Cerebrospinal Fluid Metabolomics–Lipidomics and Serine Pathway in Myalgic Encephalomyelitis/Chronic Fatigue Syndrome
Source: Int J Mol Sci. 2025 Feb 1;26(3):1282. doi: 10.3390/ijms26031282 (PMC11818353; doi:10.3390/ijms26031282)

SOM Figure S1. Pathway enrichment for Bayesian linear regression. (A) All of the significant metabolites and lipids from multivariate and Bayesian linear regression (SOM Table S1) were used for pathway enrichment using SMPDB. The top pathways are shown for A. disease, based on Figure 2; (B) gender; (C) exercise using metabolites; and (D) exercise using lipids.

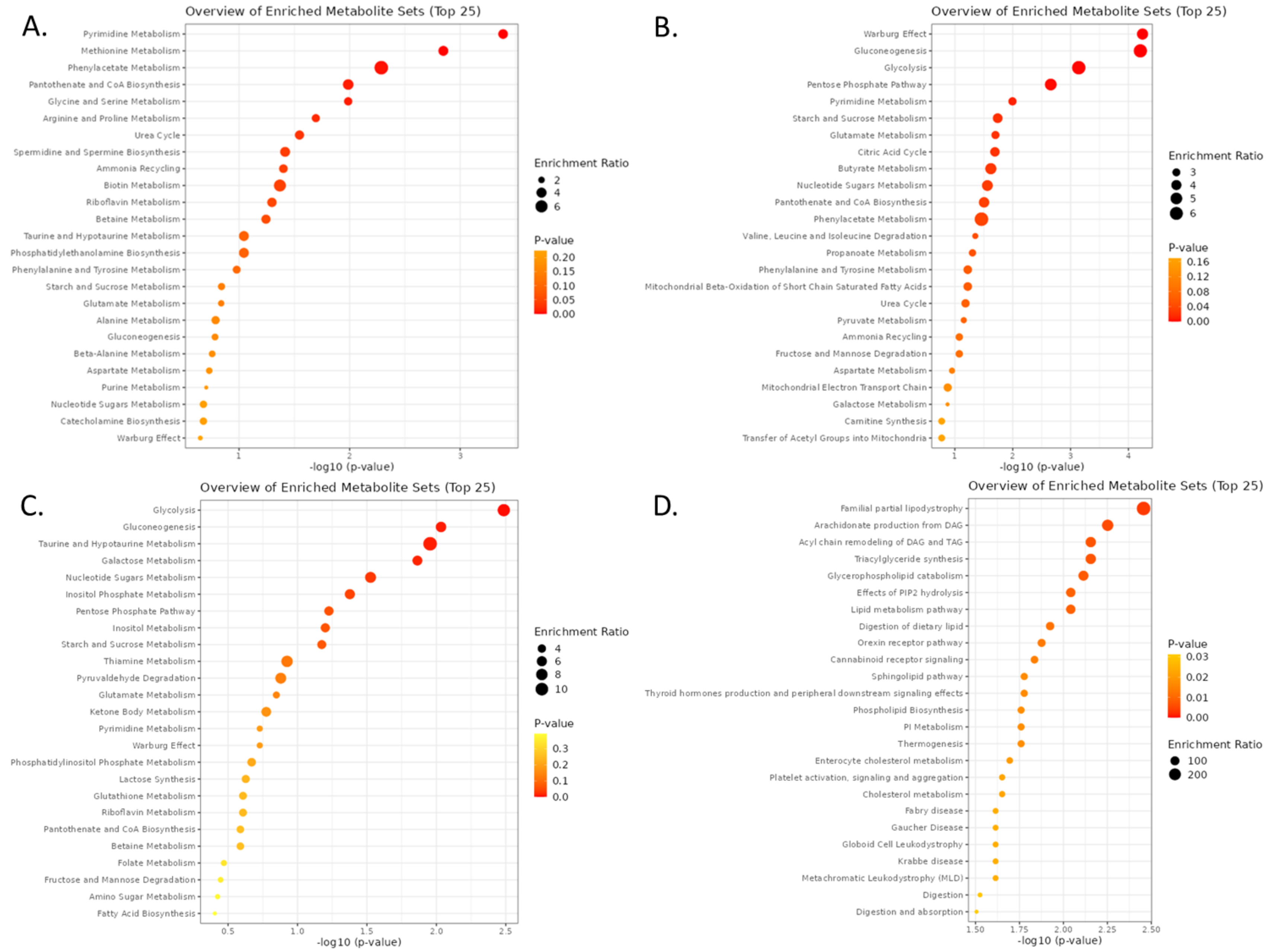

SOM Figure S2. Pathway enrichment. Metabolites that were shared between the Bayesian and multi-variate analysis were compiled and enriched for (A) non-exercise ME/CFS vs. SC, (B) ME/CFS non-exercise vs. post-exercise, and (C). SC non-exercise vs. post-exercise ($p < 0.05$ uncorrected).

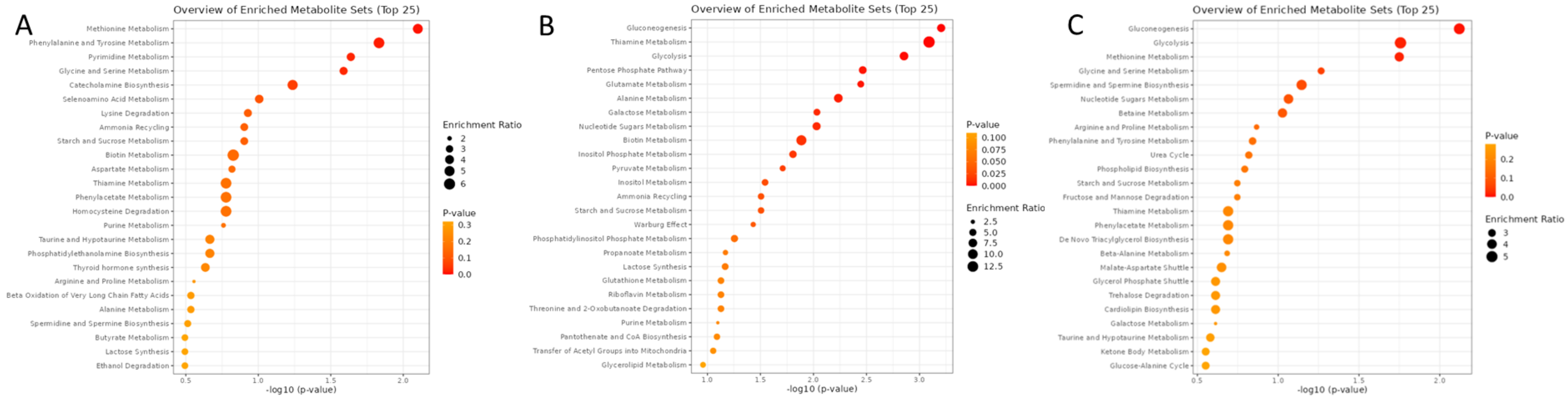

Supplement: Supplementary file 1 [file ijms-26-01282-s001.zip › ijms-3366404-supplementary.pdf]
